# Mass Suicide of COVID-19 Patient's Survivors; a Clinical Experience

**DOI:** 10.22037/aaem.v9i1.1092

**Published:** 2021-01-13

**Authors:** Mohammad Mahdi Forouzanfar, Ziba Shahini, Behrouz Hashemi, Sahar Mirbaha

**Affiliations:** 1 Department of Emergency Medicine, Shahid Beheshti University of Medical Sciences, Tehran, Iran.; 2 Department of Social Work, Shahid Beheshti University of Medical Sciences, Tehran, Iran.

**Keywords:** COVID-19, Psychological Trauma, social support, social workers

**Dear Editor**

We had just started our shift at the emergency department that day, when a highly agitated pale middle-aged woman was brought to the emergency department by the emergency medical service (EMS). Behind her were 5 of her family members, 3 were her children and the other 2 were her sister and her brother in law. She was immediately transferred to the cardiopulmonary resuscitation (CPR) room and cardiac and respiratory monitoring were provided, central venous access was established (due to lack of peripheral vascular access because of severe hypotension), and fluid infusion was performed. During the time these services were provided, a history was taken from her relatives.

The patient’s children were the ones who provided the history: apparently, their father had died 20 days before due to COVID-19. Dealing with his death was difficult for them, especially for their mother, due to the strong emotional bond with the father. The whole family had decided to commit suicide by consuming rice tablet (aluminum phosphide). On the day we met them, the mother takes an unknown number of aluminum phosphide tablets without the children knowing and then drinks instant coffee on the balcony, after which she develops symptoms and is brought to the emergency department. After a little while, the mother develops cardiorespiratory arrest in CPR, she is intubated and resuscitation is performed and her cardiac rhythm returns for 1 hour, but then develops arrest again and her heart stops beating despite cardiac resuscitation. Giving the news of her death and informing the anxious relatives waiting behind the closed doors is done with difficulty, they can be heard crying, moaning and shouting and sometimes cursing the healthcare staff.

During the second CPR, the hospital’s social worker is informed and consultation is received, especially regarding the patient’s children, and subsequently the social emergency gets involved. A few minutes later, the social worker goes to the patient’s family, explains the patient’s condition, and says that there isn’t much hope for her to return and provides consultations regarding psychological support of the family members and dangers threatening the children and emphasizes the need for involvement of the social emergency. A short while after that, the mother’s death is announced.

The social worker informs the eldest and calmest family member that each of the children should be supported by a number of family members and should not be alone at home. In addition, social emergency gets involved and sends a group of experts to take necessary measures.

From this point on, the information we will report about the patient and her family was provided by the elder daughter of the patient who is 30 years old (master of educational management): 

In the midst of the tumultuous situation and mourning and consultation with the family members, the 32-year-old son of the patient (electrical engineer), who had been very psychologically disturbed and cursed the healthcare staff after the death of his mother, leaves the emergency department for a phone call. He seemingly calls the seller of the deadly tablet and leaves the emergency department without notice. Others had thought that he is mourning somewhere in the courtyard of the hospital.

She said that her brother had left the hospital immediately after the phone call when others were receiving consultations in the hospital. After buying the tablet, he goes to his father’s grave, takes the tablet and dies there. The family members called him when they noticed that he is missing and since he did not answer, they called the police. The police found his dead body beside his father’s grave 3 hours later and informed the family. He was transferred to a hospital, which was in vain.

The younger sister who is 22 years old (master of genetics), was admitted to a mental hospital and received electroconvulsive therapy (ECT) twice during her admission. However, the older sister seems to be mournful but coping with this mental crisis.

We would like to emphasize that this family was sufficiently wealthy and highly educated from a socio-economic point of view.

The recent coronavirus epidemic had serious social and mental effects on the people of China, especially those who were quarantined and could not have face to face communications and express traditional social behaviors (1). Anxiety, fear and stress always accompany those who are affected and suspected. The families of patients with COVID-19 and people whose loved ones are in close contact with infected patients face these mental problems and this can easily turn into mass hysteria. In addition, epidemiologists, scientists, physicians, and all the healthcare professionals who are known as healthcare heroes can also be affected with these psychological problems due to involvement in this global crisis and sacrificing themselves.

Numerous studies have reported the negative psychological effects of COVID-19 including symptoms of stress due to trauma, disorientation and confusion, and anger in those involved in stressful situations. The long duration of quarantine, being afraid of getting sick, disappointment, exhaustion, storing insufficient food, incomplete information, financial problems, and stigma have been identified as stressors in the COVID-19 epidemic (2, 3). Therefore, for improvement of psychological wellbeing in Iran, people should receive psychological services and be informed of ways to access these services through various sources including social media, phone, online services, or in person.

Groups of people who might need psychological services should be identified and categorized for establishment of the connection. For instance:

Patients with COVID-19 (with mild to severe symptoms), patients whose COVID-19 test has become positive, families, friends and colleagues of the patients, all healthcare workers, researchers who study COVID-19 in laboratories, people living in regions with high risk of COVID-19, those taking care of elderly family members in their house, mothers, especially those who have 8 – 10-year-old children, pregnant women, child laborers and people who do not receive much attention, all people in any profession, patients with special mental diseases (such as obsessive compulsive disorder and mysophobia) and psychological patients who should consume medication (4).

It seems that psychological traumas due to COVID-19 are more dangerous than the pathogenic nature of the virus. This bitter clinical experience was a tragedy similar to thousands of similar incidents that might only attract the attention of a few or be neglected due to unawareness. The necessity of providing psychological support for survivors of COVID-19 patients is not less than using effective drugs for patients with COVID-19, both are lifesaving. Therefore, providing vigorous psychological support for families of these patients and not abandoning them in the face of a peculiar sorrow caused by an invisible enemy through employing social workers and social emergency teams, and establishing psychological and psychiatric consultation groups are strongly recommended and can reduce future mental disorders and suicides. The physicians are recommended to play a role by identifying these traumas, using the suggested methods, and asking for consultation and social aid for the patients’ survivors and following up on their psychological wellbeing.
